# Content and timing of feedback and reflection: a multi-center qualitative study of experienced bedside teachers

**DOI:** 10.1186/1472-6920-14-212

**Published:** 2014-10-10

**Authors:** Jed D Gonzalo, Brian S Heist, Briar L Duffy, Liselotte Dyrbye, Mark J Fagan, Gary Ferenchick, Heather Harrell, Paul A Hemmer, Walter N Kernan, Jennifer R Kogan, Colleen Rafferty, Raymond Wong, Michael D Elnicki

**Affiliations:** Department of Medicine, Pennsylvania State University College of Medicine, Hershey, Pennsylvania USA; Department of Medicine, University of Pittsburgh School of Medicine, Pittsburgh, Pennsylvania USA; Department of Medicine, University of Minnesota School of Medicine, Minneapolis, Minnesota USA; Department of Medicine, Mayo Clinic College of Medicine, Rochester, Minnesota USA; Department of Medicine, Alpert Medical School of Brown University, Providence, Rhode Island USA; Department of Medicine, College of Human Medicine, Michigan State University, East Lansing, Michigan USA; Department of Medicine, University of Florida College of Medicine, Gainesville, Florida USA; Department of Medicine, Uniformed Services University of the Health Sciences, Bethesda, Maryland USA; Department of Medicine, Yale University School of Medicine, New Haven, Connecticut USA; Department of Medicine, Perelman School of Medicine at the University of Pennsylvania, Philadelphia, Pennsylvania USA; Department of Medicine, Loma Linda University School of Medicine, Loma Linda, California USA; Division of General Internal Medicine, Penn State Hershey Medical Center – HO34, 500 University Drive, Hershey, PA 17033 USA

**Keywords:** Medical education-qualitative methods, Medical education, Medical education-faculty development, Patient centered care

## Abstract

**Background:**

Competency-based medical education increasingly recognizes the importance of observation, feedback, and reflection for trainee development. Although bedside rounds provide opportunities for authentic workplace-based implementation of feedback and team-based reflection strategies, this relationship has not been well described. The authors sought to understand the content and timing of feedback and team-based reflection provided by bedside teachers in the context of patient-centered bedside rounds.

**Methods:**

The authors conducted a thematic analysis qualitative study using transcripts from audio-recorded, semi-structured telephone interviews with internal medicine attending physicians (n= 34) identified as respected bedside teachers from 10 academic US institutions (2010–2011).

**Results:**

Half of the respondents (50%) were associate/full professors, with an average of 14 years of academic experience. In the context of bedside encounters, bedside teachers reported providing feedback on history-taking, physical-examination, and case-presentation skills, patient-centered communication, clinical decision-making, leadership, teaching skills, and professionalism. Positive feedback about physical-exam skills or clinical decision-making occurred during encounters, positive or constructive team-based feedback occurred immediately following encounters, and individualized constructive feedback occurred in one-on-one settings following rounding sessions. Compared to less frequent, emotionally-charged events, bedside teachers initiated team-based reflection on commonplace “teachable moments” related to patient characteristics or emotions, trainee actions and emotions, and attending physician role modeling.

**Conclusions:**

Bedside teachers use bedside rounds as a workplace-based method to provide assessment, feedback, and reflection, which are aligned with the goals of competency-based medical education. Embedded in patient-centered activities, clinical teachers should be encouraged to incorporate these content- and timing-related feedback and reflection strategies into their bedside teaching.

## Background

The importance of observation, feedback, and reflection for trainee development are increasingly a focus of competency-based medical education [1–3]. In 2012, the Accreditation Council for Graduate Medical Education Next Accreditation System (NAS) established educational “milestones”, or observable developmental steps that describe the trajectory of progress and educational development of trainees [4]. These observable competency-based milestones require real-time, workplace-based assessment of trainees’ skills, which include the provision of feedback and reflection across varied content areas to foster the deliberate practice needed to acquire expertise [5–9].

Although two key educational strategies required for trainee development, feedback and reflection, have been well studied, the focus has been primarily on the *process* within clinical settings. Several works identify feedback strategies used during clinical encounters and list “tips” for incorporating feedback into clinical settings [2, 10–14]. Cote and Bordage investigated the content of preceptors’ feedback in outpatient clinics, which included reading suggestions, diagnoses, patient follow-up, and residents’ concerns/feelings about cases [8]. The process of reflection facilitates the “…*analyzing, questioning, and reframing [of] an experience in order to make an assessment of it for the purposes of learning and/or to improve practice* [2, 15, 16]”. This educational method promotes both cognitive and humanistic growth, making it a necessary component of educational programs and humanistic environments [2, 17]. However, evidence suggests reflection is used little in medical education, prompting recommendations to raise awareness and use of this method [2, 16]. The literature related to both feedback and reflection establishes the conceptual framework for understanding the role of these methods in clinically-based scenarios. However, the content and timing of feedback and reflection in the context of the inpatient medicine wards are not well examined.

For internal medicine physicians-in-training while on inpatient wards, much of the authentic workplace-based action occurs during team bedside rounds – the process whereby healthcare teams provide patient-centered, point-of-care evaluation, diagnosis, and shared clinical decision-making [18–20]. Experienced medical educators and bedside teachers alike highlight the need for bedside rounds to deliver authentic assessment, feedback, and reflection [21–23]. Authenticity exists given bedside encounters allow assessment of trainees at the apex of Miller’s educational pyramid – the “does” of clinical skills [24, 25]. However, numerous barriers in hospital-based settings, including time and systems issues, limit the realization of bedside rounds [18, 26–28]. In the context NAS, a systematic investigation of how current-day bedside teachers use bedside rounds for feedback and reflection could assist in faculty development efforts geared toward competency-based education.

Through semi-structured interviews with attending physicians who perform bedside rounds, we sought to enhance understanding regarding the process and perceived benefits of bedside rounds in academic settings. Our prior publications from this project described the value, strategies for implementation, and barriers encountered during bedside rounds [22, 23, 28]. The purpose of this study was to understand the content and timing of feedback and reflection provided by bedside teachers during bedside rounds with medical students and internal medicine residents.

## Methods

### Study approach

To address the research questions and advance our understanding of bedside teachers’ strategies used in feedback and reflection during bedside rounds, a thematic analysis was used [29]. For feedback, general frameworks from Ende and Branch et al. were used during probing interview questions and initial coding [2, 14]. For reflection, although works by Branch et al. informed the understanding of the concept, no studies addressing reflection during bedside rounds were identified, therefore a data-driven, inductive approach was used [2, 16]. Semi-structured interviews were chosen rather than surveys to explore the research questions in detail. The study design and methods used in this work are described in prior publications; the *a priori* research questions investigated in this work were distinct from the other publications, which related to: (1) the value, (2) strategies for implementation, and, (3) barriers encountered during bedside rounds [22, 23, 28].

### Participant sampling

To obtain a purposive sample of institutions, one co-investigator from 10 U.S. institutions was recruited, most of whom were Clerkship Directors in Internal Medicine members or had prior research experience. Each co-investigator recruited three-six bedside teachers locally considered as bedside teachers (e.g. received bedside teaching awards, identified by faculty/residents). Each participant had to: 1) practice in general internal medicine/primary care, 2) have served as inpatient attending physician ≥2 weeks in the prior two years, and 3) perform “bedside rounds” a minimum of 3 weekdays while inpatient attending. “Bedside rounds” was defined as: “*The team of medical providers, including a minimum of one house officer and the attending physician of record, presenting the patient’s history or reviewing one physical exam component, in addition to discussing the diagnosis/management at the bedside in the patient’s presence*”. Potential participants were sent an email script by the lead investigator to obtain consent and invite them for an interview.

### Data collection

From February-November of 2010, two investigators (J.G., B.D.) performed digitally recorded, one-on-one telephone interviews, consisting of closed-ended and open-ended questions (Appendix 1). We committed to interviewing at least three participants per institution regardless if saturation was reached prior to completion of all interviews. Each recorded interview was transcribed verbatim by a professional transcriptionist. After the study, a $15 gift certificate was offered to each participant.

### Data analysis

During data collection, investigators took notes and, using the process of constant comparative analyses, identified categories and generated a preliminary codebook to facilitate analysis. The initial intent was to explore feedback strategies, however early analysis revealed participants were describing instances of reflection rather than feedback, which prompted additional code creation and modification. Two investigators (J.G., B.H.) analyzed transcripts independently with data management support from the program Atlas.ti™ 6.0 (Scientific Software, Berlin, Germany). Following independent coding of two interviews, investigators compared codes for consistency and agreement, resolved any differences by consensus, and updated the codebook. The remaining 32 interviews were coded independently, with regular adjudication sessions to modify the codebook. The technique of member checking was performed with two interviewees to support the validity of the results [30]. Lead investigators reviewed and agreed upon all themes and representative quotations. The study was exempt from further review by the Institutional Review Board at the University of Pittsburgh and each institution (Appendix 2).

## Results

Thirty-four interviews were completed, with 17 (50%) associate/full professors and 24 (71%) males, with participants averaging 14 years of academic experience. Categories and themes of feedback and reflection as they relate to bedside rounds are described below.

### Feedback

Bedside teachers observed numerous bedside activities during team rounding sessions, including conversations with patients, case presentations, physical examinations, activities related to patient-centered care, and teaching moments [22]. Based upon these observations, respondents described several areas related to feedback, including the timing, content, level of learner, direction, and overall value. The predominant descriptions, however, related to timing (outlined below and Table 1) and content (Table 1); the main categories of feedback content related to history-taking/physical-examination/case-presentation skills, patient-centered communication, clinical decision-making, leadership/teaching skills, and professionalism.

**Table 1:** **Timing, location, and content of bedside teachers’ feedback to trainees in the context of bedside rounds (n= 111 coding references)**

Timing (Location)	Frequency of code references – n (%) ^a^	Representative content discussed	Content category ^b^
During bedside encounter (bedside)	14 (13)	Insufficient physical examination performed during admission.	HCP
		Physical examination instruction or correction.	HCP
		Positive feedback on history obtained.	HCP
		Positive feedback on superior case presentation.	HCP
		Clinical reasoning or care delivered.	CDM
Immediately following bedside encounter (hallway)	48 (43)	Lengthy and wordy case presentations, with suggestions for improvement.	HCP
		Review success of bedside case presentations.	HCP
		Trainee struggling with summary statement, suggestions for improvement.	HCP
		Clinical reasoning and decision-making, with suggestions for improvement.	CDM
		Trainees not informing patient about what they are doing, e.g. physical exam.	PCC
		Trainee not speaking to comfort level of patient.	PCC
		Trainee using (in)appropriate terminology at patient level.	PCC
		Trainee hovering over patient during encounter.	PCC
		Successful patient-centered communication demonstrated by team member(s).	PCC
		Residents’ demonstration of a great teaching point at bedside.	LT
After bedside rounding sessions (private)	30 (27)	Deficiencies in note writing and history obtained.	HCP
		Missed important aspect of a patient’s past medical history.	CDM
		Medical jargon used inappropriately in front of patient.	PCC
		Trainee’s ability/deficiency to ask a patient a very sensitive question.	PCC
		Trainee’s response and way of “dealing with” an angry patient.	PCC
		Deficiencies/absence of providing student/intern feedback about presentations.	LT
		Educational skills with student/intern.	LT
		Efficiency skills in coordinating team bedside rounds.	LT
		Lack of leadership role in bedside encounter(s).	LT
		A concerning interaction or unprofessional behavior/event with a patient.	P
Mid/end-of- rotation (private)	19 (17)	Case presentations performed at bedside.	HCP
		Leadership skills in leading rounds and bedside encounters.	LT
		Assessment of core competencies on formal evaluations.	(all)

^a^Code references indicate the number of times the code was “referenced” in the analysis. For example, if feedback during the bedside encounter was discussed in detail, the code may have been referenced more than once.

^b^Content category: HCP - history-taking, case-presentation, physical-examination skills, CDM – clinical decision-making and care delivery, PCC - patient-centered communication, LT - leadership and teaching skills, P – professionalism.

Overall, bedside teachers believed bedside encounters offer numerous opportunities to observe trainees performing activities, which are unrealized without bedside rounds: “*It’s the key learning situation of the day in a case-based, patient-centered fashion*.” One attending physician summarized the message of several participants:

*“Do we use [bedside rounds] as a source for feedback? Yeah, a lot. You glean huge amounts of information about a resident, more in areas of professional behavior, interpersonal skills, management techniques, ability to lead a team more so than factual data that comes up at the bedside”.*

Another attending physician commented:

*“It’s one of the few times people are working with [trainees] directly on their clinical skills. They aren’t usually observed doing an exam or talking to patients so they don’t get specific feedback other than [the] bedside rounding situation”.*

#### During the bedside encounter

Attending physicians used time during bedside encounters as opportunities for feedback in several ways. Trainees were provided correction on physical-examination techniques (e.g. correcting stethoscope misplacement). Utilizing the bedside encounter as an opportunity for observation and feedback was exemplified in the following comment: “*If someone demonstrated a physical exam skill and there are ways that can improve, I show them in the room, in the moment*”.

Bedside encounters were used to provide feedback to students and interns about case presentations. In these instances, feedback reinforced actions done well. Some attending physicians believed positive feedback offered in patient view instills confidence in both trainee and patient: “*If it was a great presentation, I say it at the bedside. Visual confidence is helpful to patients so that they don’t feel like they have this neophyte learning doctor*”.

Several attending physicians used the bedside to provide team-based feedback about care delivered. Attending physicians highlighted how he/she would have chosen a different course or decision based upon information obtained at the bedside. For example, one participant commented:

*“A lot of my feedback is direct, more towards what I would have done differently. I do it when we are talking to the patient. We address differences we may have about the assessment or plan at the bedside so we don’t allow the patient to be confused”.*

#### Immediately following the bedside encounter

Immediately following bedside encounters outside patient rooms, many attending physicians identified the advantage of having a captive team prepared for feedback. This feedback was typically a mix of both positive and constructive content, identifying actions related to noteworthy case presentations, patient-centered communication, clinical reasoning or care delivery.

Some attending physicians believed positive feedback in a team environment is important for all team members’ education and raised expectations for feedback during subsequent encounters. One bedside teacher commented:

*“If an intern or student gave a great history or communication [skills], I do team feedback because everyone can learn from feedback even if given to one person. It has to be done correctly and people need to expect [feedback]”.*

When constructive critique was provided, content almost exclusively related to team function rather than individual performance. One participant stated: “*I comment about the quality of the encounter with the team, in the form of ‘we could have done this*”. These constructive feedback issues related to unprofessional behavior, inadequate communication, or incorrect clinical reasoning, as exemplified by one participant in the context of a delayed diagnosis:

*“We do bedside rounds, roll the patient and they’ve got an early decubitus ulcer. We make changes in their care. The point I make is the importance [of] making sure you are attending to the patient everyday and not focusing on just the problem, [but also] looking for complications”.*

Participants also highlighted the value of correcting physical examination inaccuracies:

*“When things don’t go well, I address it at that time. A third-year student presented a patient who was bacteremic and didn’t hear any murmurs. When I listened, there was no question [there was] a new murmur. We stepped out and talked about it right then. I said ‘Let’s go back in. I’ll tell the patient I want to point something out, and you need to listen again. [The murmur] wasn’t subtle”.*

#### After bedside rounding session

Following rounding sessions or later the same day, bedside teachers primarily provided individual constructive feedback in private locations. Offered to both students and residents, this feedback was less frequent than feedback provided immediately following bedside encounters.

With residents, participants focused feedback on patient-centered communication actions, efficiency, leadership, and teaching skills. If a resident used medical jargon or confusing terminology, attending physicians discussed explicitly what they observed when providing feedback. Additionally, attending physicians highlighted residents’ teaching skills managing a student/intern struggling with one aspect of bedside encounters. Similarly, in situations lacking professionalism or patient-centered care, attending physicians addressed these issues during the one-on-one private period:

*“A resident wasn’t telling the patient what he was doing. The patient said: ‘Why don’t you tell me what you are going to do before you feel my legs?.’ I talked to the resident afterwards, pointing out we need to be careful to explain everything we do to patients ahead of time and not assume they know”.*

With students and interns, attending physicians primarily discussed history-taking, case-presentation, and physical-examination skills, patient-centered communication, and clinical decision-making. Trainees struggling with case presentations, including organization, length, or developing summary statements, received feedback:

*“If [trainees] present and I see an opportunity to improve, I give suggestions. ‘You didn’t need to talk about the surgical history because it didn’t apply to this patient’s acute renal failure,’ or ‘You missed an aspect of their past medical history, which was important to why they’re here”.*

Attending physicians identified opportunities to improve communication skills, raising awareness of these instances during feedback moments:

*“I encourage them to use less jargon, speak at the comfort level of the patient, get at eye-level because they [may be] hovering over the patient, and not be afraid to color the communication with shades of good or bad, not just give objective information but also make it clear this is a favorable or concerning finding - how we feel about this finding”.*

#### Mid- or end-of-rotation

Attending physicians provided feedback at mid- and end-of-rotation sessions, focusing less on specific task-based performance and more on global evaluation. This was exemplified by the following comment: “*At the two-week point and end-of-the-rotation, I don’t talk about a specific encounter but more about how people are [performing] and ACGME competencies*”. Attending physicians provided feedback on overuse of “*facts and prognostic things*” hindering communication, or if a trainee “*really clamps up [during encounters], we talk about their discomfort*”. Lastly, attending physicians provided feedback on tasks unspecific to bedside activities (but observed at the bedside), such as how residents “ran the ship”, describing team and leadership skills.

### Reflection

Bedside teachers identified a wide range of events stimulating team-based reflection following bedside encounters, from significant, high-stakes to less poignant, low-stakes events. Significant or “seminal events”, defined by Branch et al. as events “…*that uniquely shape the values and attitudes of [trainees] who witness and participate in them, shifting the informal curriculum toward a more humanistic learning climate*”, included situations such as the breaking of bad news or the communication of a new cancer diagnosis [16]. One participant commented: “*If we have an extraordinary seminal event, [for example] if we have to break bad news, outside the room, we talk about how it went*”. These seminal events were described as infrequent occurrences. More frequently, however, attending physicians highlighted the use of “teachable moments” to stimulate team-based reflection. Less impressive than more emotionally-charged seminal events, teachable moments “…*happen that aren’t necessarily on the radar screen, but can [be] put on the radar screen*”.

Bedside teachers generally described three categories of events, or teachable moments, that triggered reflection, specifically patients’ characteristics or emotions, trainees’ actions or emotions, and attending physician role-modeling (Table 2).

**Table 2:** **General taxonomy of situations occurring during bedside encounters triggering team-based reflection (n= 47 total coding references)**

Category	Frequency of code references – n(%) ^a^	Representative examples
Patients’ characteristics or emotions	29 (62)	A patient who was emotional about his/her disease or prognosis.
		A patient who was anxious or uncomfortable about his/her diagnosis or bedside event.
		A patient who didn’t seem happy with the whole group coming to the bedside.
		A patient who didn’t seem to want to answer any questions in front of the team.
		A patient who seemed angry about an issue/event.
		A combative/“difficult” patient.
		Social aspects of the patient’s case explaining what is going on.
		Patient with “excruciating pain” but wearing make-up/eyeliner.
		Patient’s understanding of disease process/hospitalization.
		Patient’s response to breaking of bad news.
Trainees’ actions or emotions	12 (26)	Team’s incorrect diagnosis on a newly admitted patient.
		Initial bedside encounters for trainees new to the activity.
		Resident or team not acquiring an adequate history, resulting in missed diagnoses.
		Resident or team communicating the diagnosis of a new cancer to the patient.
		Resident or team communicating “bad news” to a patient.
		Resident or team response to a hostile family member.
		Resident or team demonstration of patient-centered communication skills.
		Team’s feelings regarding consulting specialist’s recommendations.
		Team’s feelings regarding event occurring at the bedside (e.g. encountering a difficult patient).
Attending physician Role modeling	6 (13)	Attending physician “setting limits” and “sticking to his guns” with a patient who acts out.
		Attending physicians clinical reasoning demonstrated at bedside.
		Attending physician’s communication skills at bedside, what went well and did not go well.
		Attending physician’s bedside demonstration of counseling a patient about his/her disease.

^a^Code references indicate the number of times the code was “referenced” in the analysis. For example, if reflection associated with a patient-related characteristic was discussed in detail, the code may have been referenced more than once.

#### Patient characteristics or emotions

Actions and responses by patients often stimulated team-based reflection. For example, upon exiting patients’ rooms, if attending physicians questioned patients’ comprehension, reflection was initiated to explore the team’s impression of the patients’ understanding. One attending physician asked the team:

*“Did you get the gut feeling the patient understood? Were you comfortable with that? Do we need to go back in and readdress it or come back this afternoon and go over more detail or assess their understanding?.”*

#### Trainees’ actions or emotions

Team-based reflection often followed changes or reaffirmation of the teams’ clinical reasoning and care delivery or discomfort, frustration, or emotions stemming from a bedside event. One attending physician commented:

*“The resident got a history on a patient with dyspnea - clearly in heart failure - and never got the history this patient had previously been diagnosed with heart failure. I don’t know if it was how she asked the questions. Things like this come up and might be a teachable moment”.*

#### Attending physician role modeling

Similarly, attending physicians initiated reflection on their own role modeling of communication or clinical reasoning. Anticipating the opportunity to reflect, attending physicians began the process prior to entering the room and completed it immediately after. The following example related to communicating a new diagnosis of cancer:

*“Let’s say we’ve diagnosed a new cancer. I’ll ask, ‘Have you ever given a patient bad news?’ If they say no, I’ll say, ‘I am going to role model this,’ or, I’ll have the resident do it. Before we go in, I’ll ‘T’ them up, ‘Watch how we go through this process.’ Then we do it, leave, and debrief. ‘How did that go?,’ ‘What did you learn?,’ ‘Is this something you can use in the future?”*

When unclear about a diagnosis, bedside teachers made the team aware of his/her own reflective processes about their diagnostic uncertainty. One attending physician commented:

*“Outside the room, we debrief: ‘Wow that was really weird. I don’t understand why this guy’s belly is so swollen when the ultrasound shows no abnormalities.’ So, I reflect on my areas of uncertainty because it’s really important to role model clinical reasoning”.*

## Discussion and conclusions

Our interviews reveal bedside teachers frequently assess actions, provide feedback, and initiate team-based reflection with trainees in the context of bedside rounds. During bedside encounters, many attending physicians provide positive feedback about history-taking, case-presentations, physical-examination skills or clinical decision-making, while immediately following bedside encounters, bedside teachers provide positive or constructive team-based feedback on skills, professionalism, and clinical decision-making. Individualized constructive feedback is offered in private, one-on-one settings after rounding sessions. Additionally, immediately following bedside encounters, bedside teachers initiate team-based reflection pertaining to socially-charged events and, more frequently, commonplace teachable moments relating to patient- or trainee-related issues. Bedside teachers use bedside rounds as a workplace-based method to provide feedback and stimulate reflection, which aligns with the prerequisites of competency-based medical education.

Nearly all participants provide feedback to trainees based on observations performed during real-time bedside encounters [6]. There are several benefits of assessment and feedback based on events occurring at the bedside. First, compared to clinically-removed assessments, these “on-the-job” events provide authentic, patient-centered in-training evaluations, which are the cornerstone of undergraduate and graduate medical education [25]. Second, trainees highly value feedback on their actions performed at the bedside, associating high-quality inpatient teaching with feedback provided on bedside skills and case presentations, notably from a credible source [25, 31]. Next, trainees most appreciate clear and accurate feedback pertaining to specific behaviors rather than undifferentiated comments about perceptions [32]. Lastly, feedback opportunities arising from team-based bedside rounds align with studies suggesting clinical performance improves with feedback focused on trainees’ needs and offered by an authoritative individual, such as an attending physician [18, 33]. Despite these recognized benefits, bedside rounds are not common practice, replaced more commonly by hallway or conference room discussions [34–36]. Likewise in medical education, studies suggest a similar shift in activities from workplace-based assessment toward non-contextually based experiences [35, 37, 38]. Without workplace-based educational methods such as bedside rounds, the “*failure(s) to obtain data or firsthand observations of a trainee’s performance*” greatly limits the quality of assessment and feedback, and subsequently, trainee development [14].

Anchored in observation and assessment of trainees during patient-centered bedside activities, the content and timing of feedback align with recommended techniques for providing high-quality feedback, which include being: well-timed, expected, regulated in quantity, based on first-hand data, and with a mutual understanding of goals between educator and trainee [2, 11, 14, 22]. Applied specifically to inpatient wards, both Irby’s bedside teachers and educators’ “tips” include debriefing immediately following bedside encounters [10–13].

The implementation of the ACGME NAS and educational “milestones” require educational models allowing for direct observation, meaningful assessment of trainee’s abilities in providing patient care, and frequent formative feedback to trainees with both significant deficiencies and more advanced skills [1, 4–6, 39]. Our exploratory analysis uncovered that bedside teachers use bedside rounds primarily as a context for near-time formative assessment and feedback, specifically related to several core ACGME core competencies, including patient care, interpersonal communication, medical knowledge and clinical reasoning, professionalism, and, through reflective exercise, practice-based learning. By focusing on patient- and trainee-centered activities, bedside teachers use the established and commonplace combined education and care-delivery method as a vehicle to achieve the prerequisites of competency-based education.

If feedback is used as a tool for the advancement of technical proficiency, then reflection leads to individual growth and maturation, both working synergistically in a trainees’ development. Attending physicians often use select bedside occurrences to initiate team-based reflection, primarily focused on everyday commonplace teachable moments rather than larger-scale and more infrequent emotionally-charged events. Although the bedside has been previously identified as a setting in which reflection could be used to foster humanism, to our knowledge, these results are the first to describe and characterize reflection strategies and the types of events leading to reflection in this setting [16, 40]. Literature suggests reflection skills are vital for professional development by promoting the analysis of an experience for the purpose of learning and can be developed by repeated guidance. Our bedside teachers’ focus of bedside events for reflection purposes spanned from cognitive-based clinical reasoning and skill development to humanistic cultivation, aligning with the previously reported “purposes” of reflection [17]. Although our study was not designed to provide an exhaustive investigation of reflection events, these results provide the foundation for subsequent work that would include developing a more comprehensive understanding of the content of bedside encounters that stimulate team-based reflection, and the quality and value of such reflection exercises for trainees, particularly in a team-based format.

Amidst current duty hour reform and pressures of inpatient medicine, these contextually-based strategies relating to the specific content and timing of feedback and reflection can be incorporated in faculty development. However, several barriers need to be addressed prior to faculty implementation. First, since current educational models and many educational milestones are realized in workplace-based contexts primarily at the patient’s bedside (both inpatient and outpatient) and with evidence suggesting feedback and reflection are enhanced by skilled mentors, supervising attending physicians are in a prime position to observe, assess, provide feedback, and stimulate reflection for trainees [41, 42]. However, many attending physicians acknowledge they do not feel equipped to give effective feedback, often fail to identify deficiencies in trainees’ clinical skills, and struggle with balancing positive and negative feedback [42–44]. Given the low prevalence of current-day bedside teaching, faculty may not only need training in assessment and feedback, but also the activity of bedside rounds [28, 39]. Second, robust assessments, feedback, and reflection require efficient processes of care, adequate staffing, time, and willingness of educators, without which task-focused trainees may be less likely to seek or be offered feedback or reflection [41]. However, as evidenced by the low prevalence of patient-centered bedside activities, inpatient wards may be a less-than-ideal environment for feedback and reflection, thereby limiting the availability and success of these opportunities [6, 45, 46]. With the implementation of the milestones and need for workplace-based experiential learning opportunities, investigations assessing the quantity and quality of feedback and reflection allowed in our current inpatient settings are required, with the goal of addressing potentially modifiable systems issues.

This study has several limitations. First, we did not have independent verification of each participants’ expertise in bedside rounds. However, each bedside teacher met the pre-specified inclusion criteria [36]. Second, our study design only allowed for the perspective of attending physicians, and therefore did not capture the perceptions of students, residents, and patients. Since interviews asked bedside teachers about their recall of activities without a validation of these reports, the results are vulnerable to recall and social desirability bias. Additionally, since only general internal medicine attending physicians were interviewed, these results may not be generalizable to subspecialty non-medicine services. Lastly, all institutions were large academic centers and these results may not be fully generalizable to smaller teaching programs.

Our study shows that bedside teachers use bedside rounds as a context for observation, feedback, and team-based reflection. Embedded in patient-centered activities, these strategies are vital for faculty development efforts, particularly in the evolving field of competency-based medical education.

### Ethical approval

Ethical approval has been granted or waived at each of the participating institutions (see Appendix 2).

## Appendix 1

Select Survey Instrument Items:

1. Close-ended survey questions:
  - What is your position in the General Internal Medicine division? (open-ended)
    i. Assistant professor
    ii. Associate professor
    iii. Professor
    iv. Chair/chief
    v. Program director/associate program director/clerkship director
    vi. Other: _______________
  - How many years have you been practicing in academic medicine? (open)
    i. _______________
  - How many weeks in the previous two years were you the “attending of record” with housestaff? (open)
    i. _______________
  - In an average week of 5 rounding days, how many days do you perform at least one bedside rounding encounter?
    i. _______________
  - During your inpatient attending time with housestaff, estimate the percentage of all patient encounters that are “bedside rounds? (open)
    i. _______________
  - Did you receive formal education about bedside rounds during the following periods in your career?
    i. Internship/residency – y/n
      If yes, in what format was this education provided?
    ii. Fellowship (if applicable) – y/n
      If yes, in what format was this education provided?
    iii. Faculty position – y/n
      If yes, in what format was this education provided?
2. Open-ended questions:
  - Why do you perform bedside rounds?
    i. Probe: Why is that? (investigate why the reason they give is important).
  - Do you debrief bedside rounding sessions?
    i. Probe: How do you debrief bedside rounding sessions?
    ii. Probe: When does this debriefing occur?
    iii. Probe: Where does this debriefing occur?
    iv. Probe: Do you debrief or provide feedback at the bedside?
    v. Probe: Can you provide a specific example?
  - Think about a successful bedside rounding encounter that you had as a teacher or learner. Please share it with me.
    i. Probe: What made the encounter successful?
    ii. Probe: What did you learn from that experience?
  - Think about an unsuccessful bedside rounding encounter that you had as a teacher or learner. Please share it with me.
    i. Probe: What made the encounter successful?
    ii. Probe: What did you learn from that experience?
  - What are the positive aspects of bedside rounds? (What are the benefits to bedside rounds?)
    i. Probe: Can you think of any additional benefits?
  - Why are bedside rounds educational for housestaff?

## Appendix 2

The participating institutions and respective Institutional Review Board (IRB) determinations involved in this work were: University of Pittsburgh School of Medicine - primary site (exempt), Alpert Medical School of Brown University (not human subjects research), Loma Linda University School of Medicine (not human subjects research), Mayo Clinic College of Medicine (minimal risk research), Michigan State Univ. College of Human Medicine (not human subjects research), Pennsylvania State University College of Medicine (exempt), Perelman School of Medicine, University of Pennsylvania (exempt), Uniformed Services University of the Health Sciences (minimal risk research), University of Florida College of Medicine (exempt), Yale University School of Medicine (exempt).
